# Osteogenic effects of electrophoretically deposited Sr-doped calcium silicate coatings on titanium

**DOI:** 10.3389/fbioe.2025.1647759

**Published:** 2025-10-09

**Authors:** Zi Wang, Yimin Du, Shunlin Zhang, Hongliang Li, Jinghong Yang, Jiyuan Yan, Zhong Li, Jinhui Liu, Juncai Liu

**Affiliations:** ^1^ Department of Orthopedics, The Affiliated Hospital, Southwest Medical University, Luzhou, China; ^2^ Sichuan Provincial Laboratory of Orthopaedic Engineering, Southwest Medical University, Luzhou, China; ^3^ Stem Cell Immunity and Regeneration Key Laboratory of Luzhou, Southwest Medical University, Luzhou, China

**Keywords:** strontium-doped calcium silicate coatings, titanium implants, electrophoretic deposition, bone tissue engineering, osseointegration, osteogenesis

## Abstract

**Background:**

Titanium (Ti) implants are mechanically reliable but lack osteoinductivity. Calcium silicate (CaSiO_3_) coatings improve bioactivity but degrade rapidly. Strontium (Sr), a bone-regulating ion, enhances osteoblast function and suppresses bone resorption. Incorporating Sr into CaSiO_3_ may synergistically improve coating stability and osteogenic performance.

**Objective:**

To develop Sr-doped CaSiO_3_ coatings with varying Sr concentrations and evaluate their effects on osteogenesis, identifying the optimal formulation for Ti surface functionalization.

**Methods:**

Sr-CaSiO_3_ powders (0.05, 0.10, 0.20 mol Sr) were synthesized by sol-gel and applied to Ti via electrophoretic deposition. The morphology and composition of the coating were analyzed using XRD, SEM, and EDS, and its effects on osteoblast-like cells (MC3T3-E1) proliferation, differentiation, mineralization, and Wnt/β-catenin pathway activation were evaluated.

**Results:**

The 0.10 mol Sr group exhibited optimal surface structure and Ca/P ratio (1.73). It significantly enhanced ALP expression, calcium nodule formation, and β-catenin nuclear translocation (p < 0.001), indicating superior osteogenic induction.

**Conclusion:**

Sr-doped CaSiO_3_ coatings enhance osteogenesis in a dose-dependent manner. The 0.10 mol Sr concentration provides the best combination of structural stability, osteoinductive capacity, and long-term bioactivity. These findings highlight the potential of Sr-doped CaSiO_3_ coatings as a promising surface modification strategy to improve the integration and clinical success of Ti implants in bone repair and regenerative medicine.

## 1 Introduction

Bone defects resulting from trauma, infection, or tumour resection remain a significant clinical challenge. Although bone tissue has a natural regenerative capacity (Salhotra et al., 2020; Global et al., 2023), this becomes insufficient once defects exceed a critical size, necessitating surgical intervention (Migliorini et al., 2021; Down et al., 2024; Yan et al., 2024; Norris et al., 2021; Yuan et al., 2024). Autologous bone grafting is the gold standard but is limited by donor-site morbidity and graft resorption, while allogeneic and xenogeneic grafts pose risks of immune rejection and infection (Wu et al., 2018; Zhang et al., 2024). These limitations have prompted the development of tissue engineering approaches integrating biomaterials, cells, and bioactive factors to promote bone repair.

Titanium (Ti) and its alloys (Ti-6Al-4V) are widely applied in orthopaedic and dental implants due to their mechanical strength, corrosion resistance, and biocompatibility (Geetha et al., 2009; Li et al., 2014; Kutty et al., 2014; Kaur and Singh, 2019; Hasan et al., 2022). However, their bioinert surfaces lack antibacterial and osteoinductive properties, often leading to infection, aseptic loosening, and eventual implant failure (Liu et al., 2004; Masters et al., 2022; Vicentini et al., 2021; Wu et al., 2020; Zhang et al., 2021). To address these issues, surface modification techniques have been employed, particularly the application of bioactive coatings that improve bone–implant integration and enhance osseointegration (Ramaswamy et al., 2009; Staiger et al., 2006; Huang et al., 2013; Kalaiva et al., 2014; Mahajan and Sidhu, 2018).

Among bioactive ceramics, calcium silicate (CaSiO_3_, Ca-Si, CS) has demonstrated excellent bioactivity and bone-bonding ability. Plasma-sprayed Ca-Si coatings significantly improve implant biocompatibility and mechanical properties and have been shown in animal studies to promote bone integration (Liu et al., 2008; Ma et al., 2014; Sadeghzade et al., 2022; Tang et al., 2021). Nevertheless, their rapid degradation in physiological environments restricts long-term stability (Mohammadi et al., 2014). To overcome this drawback, elemental doping with ions such as Zr, Zn (Li et al., 2011; Mahdy et al., 2022), Mg (Liu et al., 2022) and Ag (Palakurthy et al., 2019; Kumar et al., 2020) has been investigated, yielding composite silicates (Ca-Si-M) with improved chemical stability, adhesion strength, and in some cases antibacterial performance.

Strontium (Sr), an alkaline earth metal, occurs in human bones only as a trace element, yet it can influence bone remodelling by stimulating osteoblast activity and inhibiting osteoclast function (Hodges et al., 1950). Studies have shown that Sr activates the Wnt/β-catenin and MAPK/ERK signaling pathways, promoting osteoblast proliferation, differentiation, and mineralization. Simultaneously, Sr inhibits osteoclast activity by suppressing the NF-κB pathway (Yang et al., 2011; Zhu et al., 2016). This dual effect of “stimulating bone formation and inhibiting bone resorption” makes Sr an ideal functional ion for bone repair materials. However, the application of Sr in the body is dose-dependent, with its biological effects closely linked to concentration. At low concentrations, Sr effectively promotes bone formation and inhibits bone resorption, while high concentrations may disrupt the balance of bone calcium metabolism, potentially leading to adverse effects on bone health (Zhang et al., 2011; Marx et al., 2020; Aimaiti et al., 2017). Therefore, precise regulation of Sr concentration is essential in the design and application of Sr-modified biomaterials to achieve effective bone repair while avoiding potential toxicity.

Due to the favorable ionic radius match between Sr (0.113 nm) and Ca (0.099 nm), the calcium silicate system readily forms stable and homogeneous solid solutions (Sr_x_Ca_1-x_SiO_3_). This structure exhibits controllable Sr^2+^ release properties and remarkable crystal stability. Additionally, traditional fabrication methods, such as plasma spraying and high-temperature sintering, often lead to phase separation and lattice distortion, restricting the uniformity and functionality of the coatings (Tan et al., 2024). By contrast, the combination of the sol–gel method with electrophoretic deposition (EPD) offers both processing and functional advantages. The sol–gel route enables homogeneous Sr incorporation into the CaSiO_3_ lattice at relatively low temperatures, while EPD allows the deposition of uniform, crack-free coatings on substrates with complex geometries, with thickness and microstructure precisely controlled by adjusting voltage and time (Wu et al., 2007). Functionally, Sr^2+^ ions are released in a controlled and sustained manner, stimulating osteoblast differentiation and bone formation while inhibiting osteoclast activity, and accelerating hydroxyapatite formation in simulated body fluid (Gentleman et al., 2010; Kokubo and Takadama, 2006).

Based on these advantages, the present study synthesised Sr-doped Ca-Si powders via sol–gel processing and deposited them on Ti substrates by EPD. Coatings with different Sr concentrations (0.05, 0.10, 0.20 mol) were characterised for phase composition, morphology, and ion release, and their biological performance was assessed using MC3T3-E1 osteoblast-like cells. The aim was to clarify the influence of Sr concentration on coating properties and cell behaviour, thereby providing a rational basis for optimising Sr-doped Ca-Si coatings for Ti implants in bone repair.

## 2 Materials and methods

### 2.1 Sample preparation

Sr-doped calcium silicate (Sr-CaSiO_3_) powders were synthesised by the sol–gel method. Strontium nitrate (Sr(NO_3_)_2_, ≥99.0%, Sinopharm, Shanghai, China) and calcium nitrate tetrahydrate (Ca(NO_3_)_2_·4H_2_O, ≥99.0%, Aladdin, Shanghai, China) were used as Sr and Ca sources, respectively. Tetraethyl orthosilicate (TEOS, ≥98.0%, Macklin, Shanghai, China) served as the Si source, and nitric acid (HNO_3_, 65%–68%, Kelong, Chengdu, China) acted as catalyst. TEOS was first mixed with 2 M HNO_3_ and deionised water at a molar ratio of 1:0.16:8, stirred for 30 min to promote hydrolysis and condensation, and then Ca(NO_3_)_2_and Sr(NO_3_)_2_ were added according to the designed molar ratio of Ca(NO_3_)_2_/Sr(NO_3_)_2_/TEOS = x: (1–x): 1 (x = 0, 0.05, 0.1, and 0.2). The solution was stirred for 1 h and left standing for 2 h to form a gel, which was frozen at −20 °C for 5 h and freeze-dried for 9 h. The dried gel was calcined at 900 °C for 2 h to obtain Sr-CaSiO_3_ powders.

Commercially pure titanium (Grade 3, 1 mm thickness; Baoji XinNuo, Baoji, China) was used as the substrate. Prior to deposition, Ti sheets were polished sequentially with silicon carbide papers up to 2000 grit, ultrasonically cleaned in acetone, ethanol, and deionised water (15 min each), and air-dried. This ensured removal of surface contaminants and provided a reproducible, clean surface for deposition.

For EPD, 0.5 g of Sr-CaSiO_3_ powder was dispersed in 50 mL of anhydrous ethanol (≥99.7%, Tianjin Fuyu, Tianjin, China) and wet-milled three times (30 min per cycle). Optimised EPD conditions, determined through preliminary testing of 20–60 V and 2–10 min, were set at 40 V for 5 min with a 10 mm electrode spacing. This yielded uniform, adherent, crack-free coatings without edge effects.

### 2.2 Characterization of the physical and chemical properties of the coating surface

Phase composition was analysed by X-ray diffraction (XRD, Ultima IV, Rigaku, Japan) using Cu-Kα radiation (λ = 1.5432 Å) at 40 kV and 40 mA, scanned from 5° to 80° (2θ) at 5°/min. Peak fitting was performed with Jade 6.5 software. Surface morphology was examined by scanning electron microscopy (SEM, Zeiss, Germany), and elemental distribution was characterised using energy-dispersive X-ray spectroscopy (EDS, Oxford Instruments, United Kingdom).

### 2.3 Ion release study

Disc-shaped coated samples (8 mm diameter) with Sr contents of 0.05, 0.10, and 0.20 mol were immersed in 5 mL α-MEM (Gibco, United States) at 37 °C for 7 days. At 0.5, 1, 2, 4, and 7 days, the medium was collected and replaced with fresh α-MEM. Sr^2+^ concentrations were quantified by inductively coupled plasma-mass spectrometry (ICP-MS, Agilent 7,850, United States), and cumulative release was calculated.

### 2.4 *In vitro* formation of hydroxyapatite

The *in vitro* hydroxyapatite-forming ability of titanium sheet samples with varying strontium doping concentrations (0.05–0.20 mol) was assessed by immersion in simulated body fluid (SBF; Solarbio, Beijing, China) prepared according to Kokubo’s protocol (Kokubo et al., 1991) (pH 7.4). Samples were immersed in SBF at a concentration of 1 mg/mL and maintained in a shaking incubator at 37 °C with an agitation speed of 90 rpm for 7 days. The immersion medium was refreshed every 24 h to maintain ionic stability. Following incubation, the samples were rinsed thoroughly with distilled water and dried overnight at 60 °C to remove residual fluid. Hydroxyapatite formation on the sample surfaces was subsequently examined using SEM and EDS.

### 2.5 Cell culture

The murine osteoblast precursor cell line MC3T3-E1 Subclone 14 (CL-0378; Procell, Wuhan, China) was cultured in a 5% CO_2_ incubator at 37 °C. The culture medium was replaced every 2–3 days, depending on cell growth conditions. The basal medium was α-MEM (Gibco, United States) supplemented with 10% fetal bovine serum (FBS; Gibco, United States). For osteogenic differentiation, cells were cultured in induction medium composed of α-MEM, 10% FBS, 1% penicillin–streptomycin (Gibco, United States), 10 mM β-glycerophosphate (Sigma-Aldrich, United States), and 50 μg/mL ascorbic acid (Sigma-Aldrich, United States). When the cells reached 80%–90% confluence, they were washed once with phosphate-buffered saline (PBS; Gibco, United States) and digested with 0.25% trypsin–EDTA (Gibco, United States). After confirming even dispersion, the cells were passaged at a 1:3 ratio.

### 2.6 Cell proliferation assay

The proliferative capacity and viability of MC3T3-E1 cells in response to the samples were evaluated using a Cell Counting Kit-8 (CCK-8; Solarbio, Beijing, China). Cells were seeded in 24-well plates at a density of 1 × 10^4^ cells/well and co-cultured with the samples in a Transwell system for 1, 3, and 7 days. At each time point, the Transwell inserts containing the samples were removed, and CCK-8 solution (10% v/v in culture medium, final volume 500 μL per well) was added directly to the adherent cells in the lower chamber. After incubation at 37 °C with 5% CO_2_ for 2 h, 100 μL of the supernatant from each well was transferred to a 96-well plate, and the absorbance was measured at 450 nm using a microplate reader (Thermo Scientific, United States). Cells cultured in α-MEM supplemented with 10% FBS served as the control group.

### 2.7 Quantitative qT-PCR

Total RNA was extracted using the Super FastPure Cell RNA Isolation Kit (Vazyme, Nanjing, China) and analysed by one-step qRT-PCR with the HiScript II SYBR Green Kit (Vazyme, Nanjing, China). Data were processed according to the MIQE guidelines, and relative expression levels were calculated using the 2^−^ΔΔCt method (Livak and Schmittgen, 2001). Primers for OCN, ALP, and β-actin were obtained from NCBI reference sequences and synthesised by Sangon Biotech (Shanghai, China) (Table 1).

**TABLE 1:** Primer sequence of osteogenesis-related genes.

Gene (mouse)	Forward primer (5′→3′)	Reverse primer (5′→3′)	Accession No. (RefSeq, Mus musculus)	Amplicon (bp)
mActb	GGC​TGT​ATT​CCC​CTC​CAT​CG	CCA​GTT​GGT​AAC​AAT​GCC​ATG​T	NM_007393	154
mAlpl/Tnap (Alpl)	CCA​GAA​AGA​CAC​CTT​GAC​TGT​GG	TCT​TGT​CCG​TGT​CGC​TCA​CCA​T	NM_007431.3	110
mOCN(Bglap)	GCA​ATA​AGG​TAG​TGA​ACA​GAC​TCC	CCA​TAG​ATG​CGT​TTG​TAG​GCG​G	NM_007541.3	114

### 2.8 Western blot analysis

Total proteins were extracted on ice using RIPA lysis buffer containing protease and phosphatase inhibitors (Beyotime, China). Protein samples were mixed with loading buffer (Beyotime, China), separated on precast stain-free SDS-PAGE gels (Bio-Rad, United States) with a protein marker (Abclonal, China), and transferred to Polyvinylidene fluoride (PVDF) membranes (Merck Millipore, Germany). The membranes were incubated with primary antibodies against Runx-2 (Rabbit mAb, CST, United States) and ALP (Rabbit pAb, Abcam, United Kingdom), followed by an horseradish peroxidase (HRP)-conjugated anti-rabbit IgG secondary antibody (CST, United States). Bands were visualised using a chemiluminescence imaging system (Bio-Rad, United States). Total protein levels were quantified with Image Lab software (Bio-Rad, United States), and band intensities were analysed using ImageJ software. Protein expression was normalised to β-actin, and all experiments were performed in triplicate.

### 2.9 Alkaline phosphatase (ALP) staining and alizarin red staining

For ALP staining, the cells that underwent 14 days of osteogenic induction were fixed in 4% paraformaldehyde for 10 min and subsequently stained with BCIP/NBT staining solution (Beyotime Biotech, Shanghai, China) for 15 min. For alizarin red staining, the cells following 21 days of osteogenic induction were fixed, and then stained with 1% alizarin red S (pH 4.2) for 5 min. Stained samples were imaged using an inverted optical microscope (Olympus, Japan), and staining intensity was quantified with ImageJ software.

### 2.10 ALP activity measurement

The cells were lysed and ALP activity was measured using an assay kit (Beyotime, Shanghai, China) by reading the optical density of the cell lysates at 405 nm. Total protein content was quantified using the BCA protein quantification kit by reading the optical density at 562 nm. The enzymatic activity of ALP was normalized to the total protein content of each sample by calculating the ratio between the OD values.

### 2.11 Immunofluorescence staining

Cells were fixed with 4% paraformaldehyde for 15 min and blocked with 1% bovine serum albumin (BSA). They were incubated overnight at 4 °C with primary antibodies against Osx (1:200, Abclonal, China) and β-catenin (1:200, Abclonal, China), followed by fluorescence-conjugated secondary antibodies (anti-rabbit IgG, 1:500, Abclonal, China). After washing with PBS, nuclei were counterstained with 4′,6-diamidino-2-phenylindole (DAPI, Beyotime, China), and images were captured using a fluorescence microscope (Olympus, Japan).

### 2.12 Data analysis

All experiments were performed in triplicate. Data were analysed using GraphPad Prism (GraphPad Software, United States) and expressed as the mean ± standard deviation (SD). Statistical differences among groups were assessed by one-way ANOVA, with p < 0.05 considered statistically significant, p < 0.01 highly significant, and p < 0.001 extremely significant.

## 3 Results and discussion

### 3.1 Preparation and phase composition of Sr-Doped calcium silicate coatings

**FIGURE 1:**
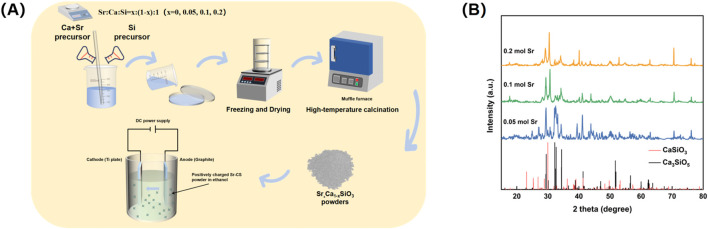
**(A)** Preparation process of Sr-doped calcium silicate coating. **(B)** XRD analysis of calcium silicate, 0.05 mol Sr, 0.1 mol Sr, and 0.2 mol Sr groups.

This study employed sol-gel EPD to fabricate a strontium-doped calcium silicate (Sr-CaSiO_3_) coating on a titanium substrate (Figure 1). As a traditional surface coating technique, the sol-gel method offers advantages such as a simple process, low material consumption, minimal equipment requirements, and no morphological constraints on the modified substrate. It has been successfully used to prepare various bioactive coatings (Abramova et al., 2020; Negrila et al., 2018). A key advantage of this method is its ability to precisely adjust the strontium doping concentration, enabling the creation of solid solution powders of calcium silicate doped with varying strontium concentrations (0.05–0.2 mol) (Sr_x_Ca_1-x_SiO_3_). To further enhance the uniformity and adhesion of the coating, EPD was combined with sol-gel to create functional coatings with varying concentration gradients on the titanium surface. EPD is a simple and rapid technique that has been successfully applied to prepare various orthopedic titanium surface coatings (Bruchiel-Spanier et al., 2022).

X-ray diffraction (XRD) analysis revealed the crystalline structural changes in the Sr-doped calcium silicate coating (Figure 1B). At a Sr doping concentration of 0.05 mol, the XRD pattern primarily showed the Ca_3_SiO_5_ phase (PDF#98-000-0043), with characteristic diffraction peak positions matching the standard reference card. This indicated that at this low doping level, Sr ions entered the calcium silicate lattice in trace amounts through an equivalent substitution mechanism, without significantly altering the matrix phase composition. Consequently, the coating retained the original Ca_3_SiO_5_ phase. However, when the Sr doping concentration increased to 0.10 mol and 0.20 mol, the XRD pattern exhibited a marked structural transition. The dominant phase shifted from Ca_3_SiO_5_ to CaSiO_3_ (PDF#98-000-0463), suggesting that as the Sr doping increased, Sr ions began to substitute for Ca, leading to the formation of a new crystal phase. Notably, the characteristic peak of CaSiO_3_ shifted by approximately 0.47° to a higher angle, which can be attributed to the larger ionic radius of Sr (1.18 Å) compared to Ca (1.00 Å). This resulted in lattice expansion or distortion, which influenced the coating’s uniformity and bioactivity, causing the diffraction peaks to shift (Collin et al., 2021; Nedunchezhian and Kannappan, 2025). The precise shift in peak position directly confirmed the role of Sr doping in modulating the lattice parameters of calcium silicate, providing quantitative evidence for the material’s crystal structure modification.

Further analysis of the XRD pattern revealed changes in the relative peak intensities. As the Sr concentration increased, the intensity of the (112) diffraction peak of Ca_3_SiO_5_ (2θ ≈ 29.8°) gradually weakened, while the intensity of the (020) diffraction peak of CaSiO_3_ (2θ ≈ 30.3°) steadily increased. These changes in doping concentration likely affected phase stability through a thermodynamic competition mechanism, promoting the transition of the crystal structure to the more stable CaSiO_3_ phase. Additionally, no diffraction peaks corresponding to SrO or other secondary phases were detected in the XRD pattern, further confirming that Sr ions entered the calcium silicate lattice via the equivalent substitution mechanism (Querido et al., 2014; Querido et al., 2016), rather than forming independent secondary phases. This finding ensures the structural uniformity and stability of the coating.

### 3.2 Morphology of Sr-doped calcium silicate coating

**FIGURE 2:**
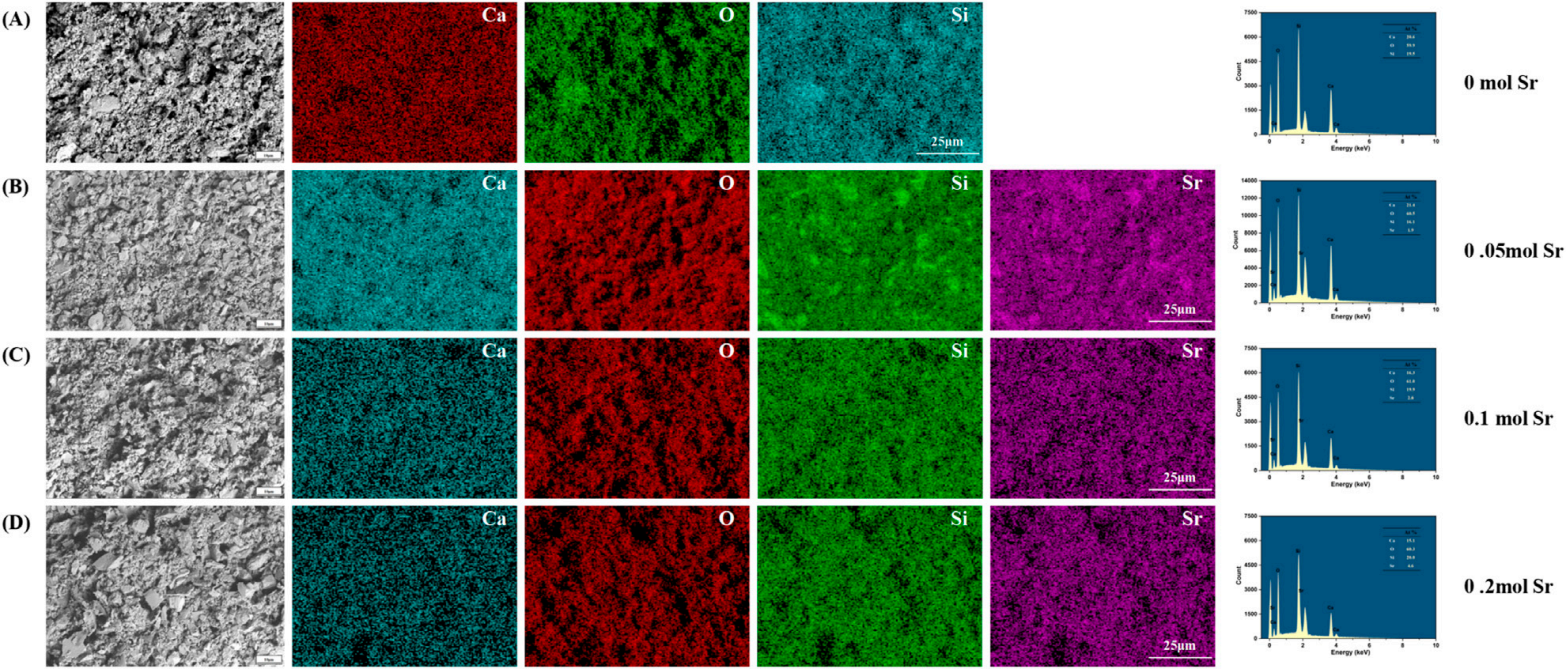
Surface microtopography by SEM and EDS analysis. **(A)** 0 mol Sr group; **(B)** 0.05 mol Sr group; **(C)** 0.1 mol Sr group; **(D)** 0.2 mol Sr group.

Scanning electron microscopy (SEM) coupled with energy dispersive X-ray spectroscopy (EDS) analysis revealed the microstructure and elemental distribution characteristics of calcium silicate coatings at different Sr-doping concentrations (Figure 2). In the control group with no Sr doping (0 mol Sr group, Figure 2A), the coating surface consisted of irregular particles ranging from submicron to micron size, loosely stacked with noticeable porosity between the particles. EDS spectra showed atomic percentages of 59.9% for oxygen (O), 20.6% for calcium (Ca), and 19.5% for silicon (Si), with no detectable Sr signal. This indicates that the chemical composition is consistent with pure calcium silicate (Ca_3_SiO_5_). Although the porous structure promotes ion diffusion, the limited number of active surface sites may restrict the efficiency of biological mineralization.

As the Sr doping concentration increased to 0.05 mol (Figure 2B), the microstructure of the coating underwent significant changes. Large particles (4–10 μm) in the form of plates or blocks were embedded in the existing micron/submicron particle network. EDS results showed that Sr comprised 1.9% of the coating, with slight fluctuations in the proportions of O (60.5%), Ca (21.4%), and Si (16.1%) compared to the undoped group, suggesting that Sr was uniformly solid-solved into the lattice by substituting Ca. No significant phase separation was observed. The relatively large ionic radius of Sr (1.18 Å) may induce local lattice distortions, promoting anisotropic growth of CaSiO_3_ crystals and forming composite particle structures, which enhance the specific surface area and surface activity of the coating.

At a Sr concentration of 0.1 mol (Figure 2C), the particle morphology evolved into a more uniform, near-spherical structure, with a nanofiber-like “fuzz” covering the particle surfaces. EDS analysis showed a slight increase in the oxygen atomic percentage to 61.0%, while the proportions of Ca (16.3%) and Si (19.9%) were significantly adjusted, and the Sr content rose to 2.8%. The formation of this nanofiber structure is likely due to lattice-stress-driven surface reconstruction. Its high surface area and oxygen-rich characteristics may synergistically promote the nucleation of hydroxyapatite (HA) precursors, providing more active sites for biological mineralization.

When the Sr concentration increased to 0.2 mol (Figure 2D), larger particles and pore defects appeared in the coating. At this stage, the oxygen atomic percentage was 60.3%, while the ratios of Ca (15.1%) and Si (20.0%) decreased further, and Sr content increased to 4.6%. Excessive Sr doping may cause a kinetic imbalance, leading to particle aggregation and the formation of pores. Despite the higher oxygen enrichment, structural inhomogeneity may weaken the continuity of ion release channels, limiting the mechanical stability and uniformity of the bioactive layer in the coating.

A comparison of the results at different Sr concentrations showed that the 0.10 mol Sr-doped group achieved an optimal balance between morphology (near-spherical particles and nanofiber structures) and chemical composition (O: 61.0%, Sr: 2.8%). The high density of active sites on its surface, along with its oxygen-rich characteristics, can synergistically enhance its biological mineralization potential, providing a solid foundation for subsequent cell behavior regulation. In contrast, although the 0.20 mol group had a higher Sr content, the presence of structural defects partially negated the advantages of the chemical composition, highlighting the importance of precise Sr concentration control during coating preparation.

### 3.3 Formation of apatite on Sr-Doped calcium silicate coated titanium substrates

**FIGURE 3:**
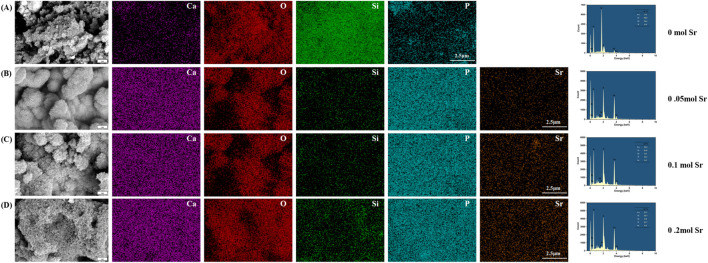
SEM and EDS Characterization of Surface Microstructure After One-Week SBF Soaking in Different Groups. **(A)** 0 mol Sr group; **(B)** 0.05 mol Sr group; **(C)** 0.1 mol Sr group; **(D)** 0.2 mol Sr group.).

To evaluate the biomineralization behavior of calcium silicate coatings with different Sr doping concentrations, samples were immersed in simulated body fluid (SBF) for 7 days and characterized using SEM and EDS (Figure 3). In the coating without Sr doping (0 mol, Figure 3A), EDS analysis showed atomic percentages of 58.5% O, 4.3% Ca, 36.3% Si, and 0.9% phosphorus (P), resulting in a Ca/P ratio of 4.78. This ratio is significantly higher than the ideal value for hydroxyapatite (HA, Ca/P ≈ 1.67), indicating that the surface is primarily composed of a calcium-rich phase (such as calcium carbonate or amorphous calcium phosphate) with minimal HA deposition. Furthermore, the particles exhibited irregular shapes and considerable aggregation, suggesting relatively low biological activity. The high Ca/P ratio could be due to the silicate network (Si = 36.3%) hindering phosphate ion adsorption, or local calcium enrichment in the EDS measurement areas, potentially introducing measurement bias. This hypothesis should be further verified through statistical analysis with multiple sampling points.

When the Sr doping concentration increased to 0.05 mol (Figure 3B), the Ca/P ratio improved significantly to 1.73 (Ca: 25.4%, P: 14.7%), approaching the ideal ratio for HA. Simultaneously, the Si content decreased drastically to 0.1%, likely due to Sr introduction, which promoted the ordered deposition of calcium phosphate through surface charge modification. This effect likely led to the covering or partial dissolution of the silicate phase by phosphate. Microscopic examination revealed a change in particle morphology from irregular aggregates to near-spherical structures, with a nanofiber-like “fluff” on the surface, indicating preferential nucleation of HA precursor phases. However, the abrupt reduction in Si content appears inconsistent with the stability of the matrix phase (Ca_3_SiO_5_) observed by XRD, further supporting the hypothesis that the silicate phase was either covered or dissolved through surface interactions. While EDS primarily analyzes elemental composition at selected points, lines, or areas, XRD reflects the overall crystalline structure stability and phase composition of the prepared material.

In the 0.1 mol Sr group (Figure 3C), the Ca/P ratio stabilized at 1.73 (Ca: 24.4%, P: 14.1%), and the Si content slightly increased to 0.3%. This suggests that Sr doping effectively suppressed the silicate phase and promoted stable deposition of phosphate. The surface nanofiber-like structure also enhanced the specific surface area and nucleation site density. The minor Sr incorporation (0.3%) likely optimized the surface charge distribution through polarization effects, further improving HA deposition efficiency. In contrast, the 0.2 mol Sr sample (Figure 3D) exhibited a slight decrease in the Ca/P ratio to 1.71 (Ca: 21.7%, P: 12.7%) and a small increase in Si content (0.5%). This could be attributed to residual silicate or measurement errors in specific areas, although the overall trend still suggests Sr’s inhibitory effect on silicate formation. Additionally, the oxygen percentage peaked at 64.7%, accompanied by noticeable structural defects, such as pores and particle agglomeration. These defects may disrupt the uniformity of HA deposition, indicating that excessive Sr doping (0.2 mol) may lead to kinetic imbalances that require careful control during the coating preparation process.

The 0.05–0.1 mol Sr-doped groups significantly enhanced the biomineralization ability of the coatings by optimizing both the Ca/P ratio (approaching 1.67) and morphology (formation of nanofibers). These results provide an important concentration range for the design of coating materials in bone tissue engineering applications.

### 3.4 Surface roughness and Sr^2+^ release behaviour

**FIGURE 4:**
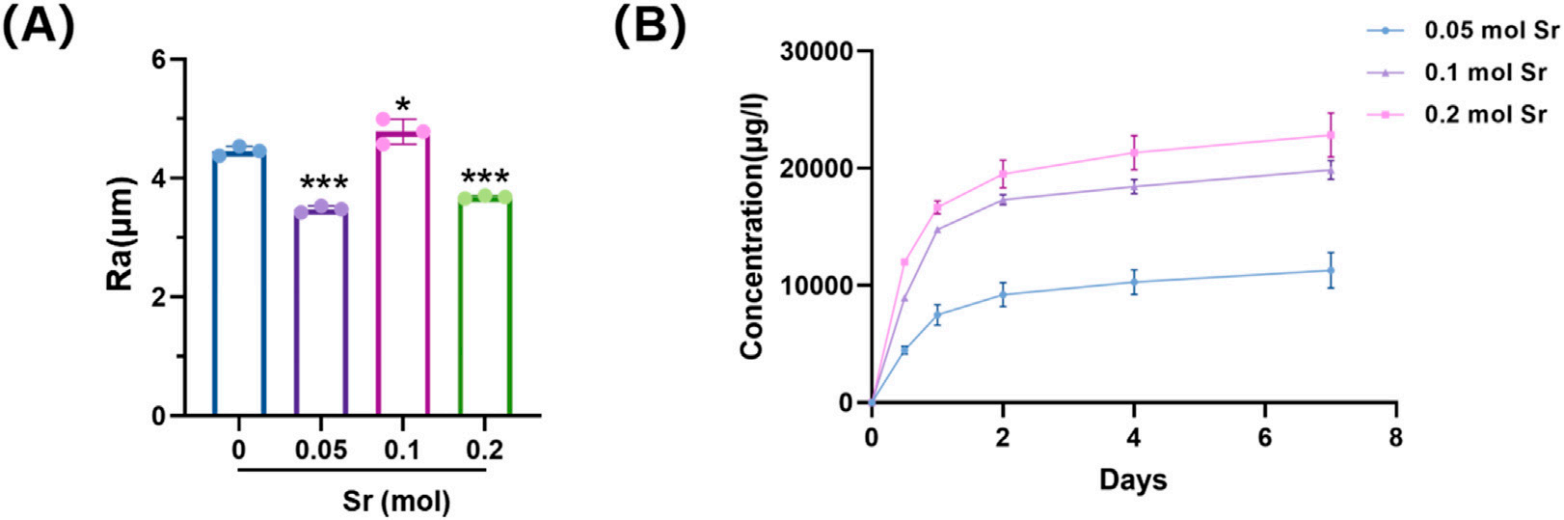
Surface roughness and Sr^2+^ release of Sr-doped CaSiO_3_ coatings. **(A)** Average surface roughness (Ra) values of coatings with different Sr doping concentrations. **(B)** Cumulative Sr^2+^ release profiles over 7 days.

Sr doping altered both the surface roughness and ion release characteristics of the coatings (Figure 4). The 0.10 mol Sr group exhibited a moderate Ra value, reflecting a uniform and stable surface morphology that facilitated active site formation. This structural feature was accompanied by a biphasic Sr^2+^ release profile, consisting of an initial rapid release during the first 2 days followed by a sustained release phase up to day 7. Such a pattern provides sufficient ionic stimulation for early osteogenic activation while maintaining long-term bioactivity to support differentiation and mineralisation. By contrast, the 0.05 mol Sr group displayed lower roughness and reduced ion release, whereas the 0.20 mol group showed excessive roughness with surface defects, leading to accelerated release and potential instability. Thereby optimising osteogenic outcomes.

### 3.5 Different concentrations of Sr-doped calcium silicate coatings can promote osteogenesis *in vitro*

**FIGURE 5:**
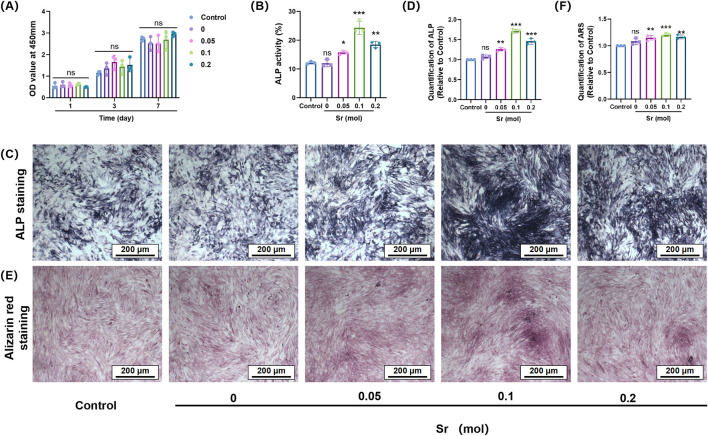
The effect of Sr-doped calcium silicate coatings with different concentrations on osteogenic differentiation of MC3T3-E1 cells. **(A)** The effect of materials on the viability of MC3T3-E1 cells; **(B)** Quantitative analysis of ALP activity of MC3T3-E1 cells cultured with different concentrations of materials for 7 days; **(C,D)** Representative ALP staining images of MC3T3-E1 cells after 7 days of culture and the quantitative analysis; **(E,F)** ARS staining images of MC3T3-E1 cells cultured with materials of different concentrations for 21 days and the quantitative analysis of mineralised calcium nodules. (nsP>0.05 is considered not statistically significant; *p < 0.05; **p < 0.01; ***P < 0.001 is considered statistically significant).

In the cellular functional study (Figure 4), coatings without strontium doping exhibited relatively weak osteogenic activity. However, the introduction of Sr significantly altered this outcome, demonstrating a clear concentration-dependent regulatory effect. CCK-8 cell viability assays (Figure 5A) indicated that Sr doping did not significantly affect the proliferation rate of osteoblast-like cells (MC3T3-E1), suggesting that the coating system possesses good biocompatibility and that the release of Sr does not exert toxic effects on cell metabolic activity. ALP staining results (Figures 5B–D) revealed a noticeable increase in ALP activity as the Sr concentration increased from 0.05 mol to 0.10 mol, with a significant enlargement of the stained area. This trend indicates enhanced activation of osteogenic differentiation in the early stages, with increasing Sr concentrations. The 0.10 mol Sr-doped group exhibited the highest ALP activity, showing optimal regulation during the early stage of osteogenesis. However, when the Sr concentration was further increased to 0.20 mol, the ALP activity slightly decreased due to excessive particle aggregation and pore formation on the coating surface, although it remained significantly higher than the undoped group. In late-stage mineralization, ARS staining and quantitative analysis (Figures 5E,F) confirmed this trend. The 0.10 mol Sr group showed uniformly distributed and dense calcium nodules, exhibiting the most prominent mineralization ability. Although the 0.20 mol Sr group displayed some sparse deposition due to structural defects, its overall mineralization level was still significantly superior to the undoped group.

Strontium doping not only did not inhibit cell growth but also effectively promoted the transition of cells from the proliferation stage to the differentiation stage by releasing functional ions. Specifically, the 0.10 mol Sr-doped concentration achieved a balanced interplay between biological activity and ion stimulation, further validating the importance of the dose-dependent effect in the design of bone tissue engineering materials.

**FIGURE 6:**
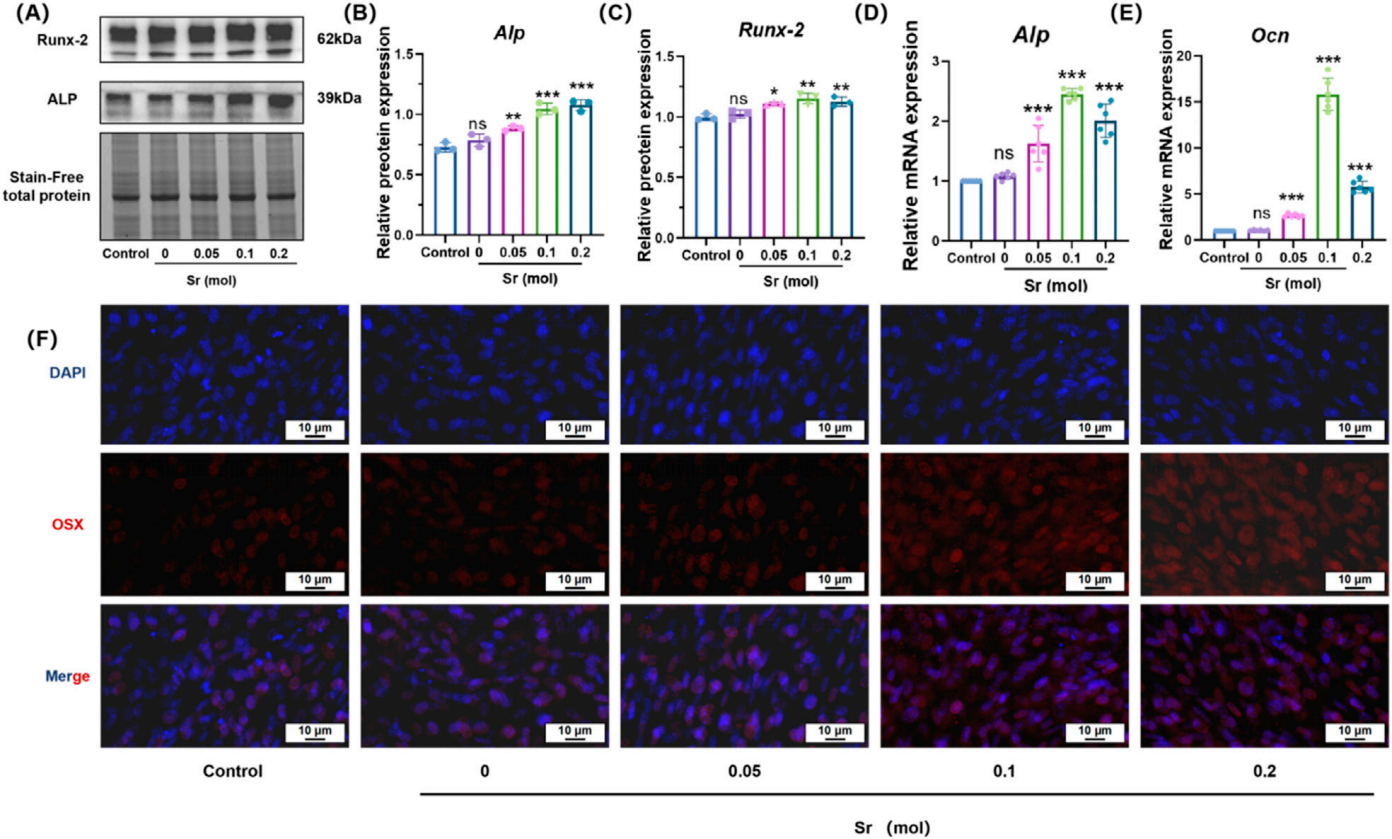
Osteogenic differentiation of MC3T3-E1 cells induced by Sr-doped materials, assessed through protein expression, mRNA levels, and OSX immunofluorescence. **(A)** Effects of materials containing different concentrations of strontium on osteogenic protein expression levels in MC3T3-E1 cells. **(B,C)** Protein quantification levels of RUNX2 and ALP. **(D,E)** PCR was used to detect the mRNA expression of OB specific markers ALP and OCN. **(F)** Representative images of immunofluorescence staining after co-culturing MC3T3-E1 cells with materials of different concentrations of strontium using OSX (red) and DAPI (blue) for 7 days. Scale bar = 10 μm. (^ns^P > 0.05 is considered not statistically significant; *p < 0.05; **p < 0.01; ***P < 0.001 is considered statistically significant.).

To investigate the molecular mechanisms underlying Sr-doping-induced osteogenic differentiation, a systematic study of relevant signaling pathways and gene expression was conducted using techniques such as Western blot, qPCR, and immunofluorescence (Figure 6). Western blot analysis (Figure 6A) revealed a significant increase in the expression levels of osteogenic markers ALP (Figure 6B) and Runx-2 (Figure 6C) with increasing Sr doping, with the highest expression observed in the 0.10 mol Sr group. The qPCR results (Figures 6D,E) were highly consistent with the Western blot findings, further confirming that Sr doping not only enhanced the gene expression of early osteogenic markers (Alp) but also significantly increased the transcription levels of late-stage mineralization proteins, such as Ocn. These results suggest that Sr plays a pivotal role throughout the entire osteogenic differentiation process, from the initiation of early cell differentiation to the formation of mineralized nodules in the later stages, exerting a positive regulatory effect at each stage.

### 3.6 Sr enhances β - catenin nuclear translocation

**FIGURE 7:**
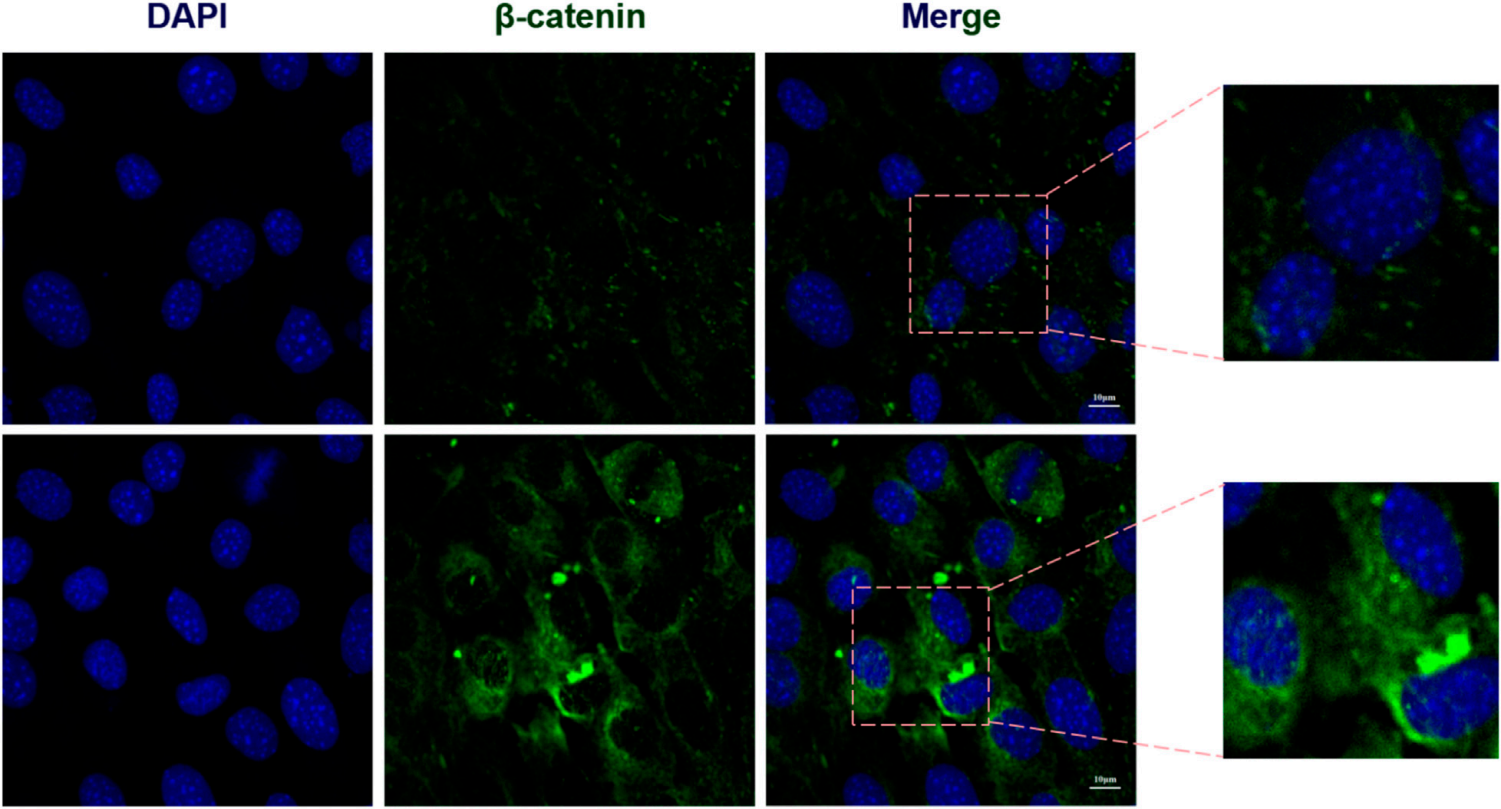
Sr enhances β-catenin nuclear translocation. Immunofluorescence analysis of Sr-induced β-catenin nuclear translocation.

Immunofluorescence images provided visual evidence confirming that Sr doping significantly enhanced the nuclear localization of OSX (an osteoblast-specific transcription factor) (Figure 5F), as well as the nuclear translocation of β-catenin (Figure 7). Compared to the control group, the 0.10 mol Sr group exhibited a marked increase in β-catenin nuclear translocation, with stronger red fluorescence signals of β-catenin observed within the cell nucleus. In contrast, β-catenin in the control group was predominantly localized in the cytoplasm. These results suggest that the Wnt/β-catenin signaling pathway plays a critical regulatory role in Sr-induced osteogenic differentiation.

Research on surface modifications of titanium and titanium alloys to improve the integration of implants with bone tissue has become a key focus in orthopedics (Walaa et al., 2024; Ying and Chang-yi, 2013). In particular, HA coatings, with a chemical composition similar to natural bone tissue, effectively promote bone integration between the implant and host bone, thereby extending the implant’s lifespan (Arcos and Vallet-Regí, 2020). However, the low bonding strength between HA and the titanium substrate has led to suboptimal long-term clinical outcomes (Otten et al., 2016). In contrast, calcium silicate ceramics exhibit high bonding strength with titanium substrates, and their coefficient of thermal expansion (CTE) is comparable to that of Ti6Al4V (Wu et al., 2009), demonstrating excellent adhesion strength. Additionally, Ca^2+^ and Si^2+^ ions released from calcium silicate can enhance osteoblast adhesion and proliferation, thus promoting osteogenic differentiation (Cao et al., 2023).

To further enhance the osteoinductive potential of the coating, doping ions into the coating mimics trace elements found in natural bone tissue, which play important roles during bone development and maturation. However, in this study, undoped pure calcium silicate (CaSiO_3_) coatings did not exhibit significant osteogenic effects, which deviates from the common understanding that calcium silicate materials generally possess bioactivity (Wang et al., 2015; Xue et al., 2005). The surface defects of the material (Figure 2A) may be a primary factor contributing to the insufficient osteogenic effect of the coating. The surface morphology of the undoped group showed irregularly aggregated particles and higher porosity but lacked sufficient active sites, which may have limited cell attachment and proliferation. More importantly, the rapid degradation rate and porous structure of calcium silicate accelerated the release of Ca^2+^nd SiO_4_^4-^ ions, and the transient high concentration of these ions might have caused a sudden increase in the local microenvironment’s pH or ionic imbalance, inhibiting cell activity (Iimori et al., 2005; Ramaswamy et al., 2008).

The weak bio-mineralization ability of calcium silicate (Figure 3A) could be attributed to the high calcium-to-phosphorus ratio (Ca/P ≈ 4.78) formed by the material in SBF, which is much higher than the ideal ratio for HA (Ca/P ≈ 1.67). This suggests that a high-silica environment may hinder effective bio-mineralization and reduce the coating’s bioactivity (Wang et al., 2016). Moreover, this study has not yet explored the impact of strontium doping on the mechanical properties of the material. Previous studies have shown that the surface properties of implants, including porosity, roughness, and shape, play crucial roles in regulating cell growth, adhesion, and protein binding (Vijayakumar and Swamiappan, 2022). Specifically, the irregularity of rough surfaces aids in the nucleation of mineral deposition, promoting HA growth, and positively influencing osteoblast proliferation and extracellular matrix production, which are essential for bio-mineralization (Nedunchezhian and Kannappan, 2025; Choudhary et al., 2016).

Despite the promising findings, several limitations of this study should be acknowledged. First, although MC3T3-E1 cells are widely used as an osteoblast-like model, they are in fact murine fibroblast-derived cells (ATCC) and thus do not fully recapitulate the phenotype and behaviour of primary osteoblasts *in vivo* (Hwang and Horton, 2019). Accordingly, all results should be interpreted as indicative rather than conclusive, and further validation in human primary osteoblasts and animal models is required. Second, while our *in vitro* assays demonstrated enhanced osteogenic differentiation, they cannot reliably predict the complex biological responses within the dynamic bone microenvironment. As highlighted by Bohner (Bohner and Lemaitre, 2009), SBF tests and cell-based assays are only partial predictors of *in vivo* bioactivity, and their limitations must be carefully considered when extrapolating to clinical applications. Third, although Sr incorporation improved bioactivity, its effect on mechanical properties, coating adhesion under load, and long-term degradation was not evaluated in this work, which may influence clinical reliability. Finally, the antimicrobial performance, vascularisation potential, and immunomodulatory effects of Sr-doped coatings remain unexplored, yet these aspects are critical for translation. Taken together, these limitations provide context to our conclusion: while the 0.10 mol Sr-doped CaSiO_3_ coating showed the most favourable osteogenic profile *in vitro*, comprehensive *in vivo* studies are indispensable to confirm its safety, mechanical stability, and long-term efficacy for clinical application.

## 4 Conclusion

Sr-doped CaSiO_3_coatings were fabricated on titanium substrates by sol–gel-assisted electrophoretic deposition, yielding uniform morphology, controlled Sr incorporation, and favourable bioactivity. Owing to their sustained ion release, the 0.10 mol Sr formulation achieved the most balanced biological performance, with pronounced enhancement of ALP expression, mineralisation, and osteogenic signalling. These results indicate that precise regulation of Sr concentration can mitigate the intrinsic lack of osteoinductivity in titanium and provide a promising avenue for bone repair. To progress from *in vitro* validation to clinical translation, however, further studies must address several critical issues: *in vivo* confirmation of osteogenic efficacy and biosafety, long-term mechanical stability under physiological loading, and the integration of additional functionalities such as antimicrobial activity and pro-angiogenic potential. Moreover, elucidating the interactions of Sr with osteoblasts, osteoclasts, and the immune environment will be essential for the rational design of next-generation ion-doped coatings with both biological efficacy and clinical reliability.

## Data Availability

The original contributions presented in the study are included in the article/supplementary material, further inquiries can be directed to the corresponding authors.
